# Integrative Multi-Omics Analysis Reveals the Molecular Characteristics, Tumor Microenvironment, and Clinical Significance of Ubiquitination Mechanisms in Lung Adenocarcinoma

**DOI:** 10.3390/ijms26136501

**Published:** 2025-07-06

**Authors:** Deyu Long, Yajing Xue, Xiushi Yu, Xue Qin, Jiaxin Chen, Jia Luo, Ketao Ma, Lili Wei, Xinzhi Li

**Affiliations:** 1 The Key Laboratory of Xinjiang Endemic and Ethnic Diseases, Ministry of Education, Shihezi University Medical College, Shihezi 832000, Chinamaketao@shzu.edu.cn (K.M.); 2 Department of Physiology, Shihezi University School of Medicine, Shihezi 832003, China

**Keywords:** lung adenocarcinoma, multi-omics, ubiquitination regulators, ubiquitination, immunotherapy

## Abstract

Ubiquitination is a dynamic and reversible post-translational modification mediated by ubiquitination regulators (UBRs), which plays an essential role in protein stability, cell differentiation and immunity. Dysregulation of UBRs can lead to destabilization of biological processes and may induce serious human diseases, including cancer. Many UBRs, such as E3 ubiquitin ligases and deubiquitinases (DUBs), have been identified as potential drug targets for cancer therapy. However, the potential clinical value of UBRs in lung adenocarcinoma (LUAD) remains to be elucidated. Here, we identified 17 hub UBRs from high-confidence protein–protein interaction networks of UBRs correlated with cancer hallmark-related pathways using four topological algorithms. The expression of hub UBRs is affected by copy number variation and post-transcriptional regulation, and their high expression is often detrimental to patient survival. Based on the expression profiles of hub UBRs, patients can be classified into two ubiquitination subtypes with different characteristics. These subtypes exhibit significant differences across multiple dimensions, including survival, expression level, mutation burden, female predominance, infiltration level, immune profile, and drug response. In addition, we established a scoring system for evaluating the ubiquitination status of individual LUAD patients, called the ubiquitination-related risk (UB_risk) score, and found that patients with low scores are more likely to gain advantages from immunotherapy. The results of this study emphasize the critical role of ubiquitination in the classification, tumor microenvironment and immunotherapy of LUAD. The construction of the UB_risk scoring system lays a research foundation for evaluating the ubiquitination status of individual LUAD patients and formulating precise treatment strategies from the ubiquitination level.

## 1. Introduction

Lung cancer is the leading cause of cancer-related mortality and one of the most frequent malignant tumors worldwide. Among them, lung adenocarcinoma (LUAD) originating from small airway epithelial cells, glandular cells, and/or type II alveolar cells is the main pathological subtype of lung cancer and has significant histological heterogeneity [1,2,3]. LUAD is often locally advanced or metastatic at the time of diagnosis due to its indolent clinical presentation and location at the margins of the lung parenchyma [4]. Over the past decade, LUAD treatments such as surgery, chemotherapy, radiotherapy, targeted therapy, and immunotherapy have progressed, greatly improving early-stage patient prognosis. However, advanced LUAD prognosis remains poor [5,6]. Omics data can provide multidimensional, high-resolution molecular information, which helps to identify the molecular characteristics and tumor subtypes of LUAD [2]. Therefore, comprehensive and in-depth characterization of LUAD patients can be achieved by integrating tumor genomics, transcriptomics, proteomics, and clinical data. This characterization can offer new perspectives for the precise diagnosis and treatment of LUAD patients.

Ubiquitination is a pivotal and reversible post-translational modification of proteins that plays an indispensable role in regulating protein stability, cell survival, cell differentiation, innate and adaptive immunity [7,8]. Ubiquitination regulators (UBRs) regulate the dynamic reversible process of ubiquitination, and are divided into three groups according to their functional categories: writers, readers, and erasers. Specifically, E1 ubiquitin-activating (E1) enzymes, E2 ubiquitin conjugating (E2) enzymes, and E3 ubiquitin-ligating (E3) enzymes are writers, which are responsible for adding ubiquitin molecules to substrate proteins. Proteins containing ubiquitin and ubiquitin-like binding domains (UBDs) or ubiquitin-like domains (ULDs) are readers. They are responsible for recognizing ubiquitination modifications and linking ubiquitination substrates to downstream events, including protein degradation, relocalization, formation of multiprotein complexes, activation of enzymatic pathways, and other events. Deubiquitinases (DUBs) are erasers, which are responsible for removing ubiquitin from substrate proteins [9,10,11,12,13]. Altered biological processes when ubiquitination regulatory mechanisms are aberrant may induce serious human diseases, especially various types of cancer. The biological processes that are altered during tumorigenesis include tumor metabolism, tumor microenvironment, and cancer stem cell stemness. For example, in terms of tumor metabolism, the ubiquitination of key molecules such as RagA, mTOR, AKT, c-Myc, PTEN, and P53 significantly regulates the activity of mTORC1, AMPK, and PTEN-AKT signaling pathways [14]. In addition, some UBRs (such as E3s and DUBs) are potential drug targets for anti-cancer therapeutic strategies, and many small-molecule inhibitors targeting UBRs have been developed, such as MLN4924 (targeting the E1 enzyme), Leucettamol A (targeting the E2 enzyme), nutlin (targeting the E3 enzyme), and compound G5 (targeting DUB activity) [14,15,16,17]. Therefore, systematic investigation of the role of UBRs in LUAD will help us to understand the occurrence and development of LUAD from the ubiquitination level and promote the development of therapeutic strategies focusing on ubiquitination.

The tumor microenvironment is a complex environment in which tumor cells survive and develop, including surrounding immune cells, fibroblasts, inflammatory cells, blood vessels, various signaling molecules, and extracellular matrix. The communication between tumor cells and the tumor microenvironment is not only directly responsible for the occurrence, development, and metastasis of tumors, but is also intimately connected with the treatment outcome and long-term prognosis of patients [18,19]. Immune checkpoints consist of inhibitory and stimulatory pathways and are important immunomodulators for maintaining immune homeostasis and preventing autoimmunity. They are essential for maintaining self-tolerance and regulating the type, intensity, and duration of immune responses. CTLA-4, PD-1, and PD-L1 are the most widely studied and recognized inhibitory checkpoint pathways, and drugs that block these pathways have been used to treat a variety of malignancies [20]. Toll-like receptors, RIG-like receptors, and DNA recognition receptor signaling pathways are critical in the immune system, and their ubiquitination modulates the tumor microenvironment [14]. Therefore, constructing the immune landscape of UBRs in LUAD may provide new perspectives for the classification and treatment of LUAD patients.

Here, the activities of 28 cancer hallmark-related pathways were found to be significantly correlated with the expression of 79 UBRs, and 17 hub UBRs were identified from the filtered PPI network by four topological algorithms. Hub UBRs have widespread genetic alterations and expression perturbations in LUAD, and their high expression is usually detrimental to patient survival. Based on the expression matrix of hub UBRs, a consensus clustering method was used to divide LUAD patients into two ubiquitination subtypes with significant differences in survival rate, gender ratio, mutation burden and immune characteristics. Moreover, a scoring scheme named UB_risk (ubiquitination-related risk) was constructed based on the expression profile of hub UBRs to evaluate the ubiquitination status of individual LUAD patients, revealing that patients with low-UB_risk scores are more likely to gain advantages in immunotherapy. In summary, this study aims to explore the ubiquitination status of patients with LUAD through UBRs, which can provide guidance for formulating precision therapeutic strategies for LUAD patients from the ubiquitination layer.

## 2. Results

### 2.1. Recognition of Hub UBRs

**Figure 1:**
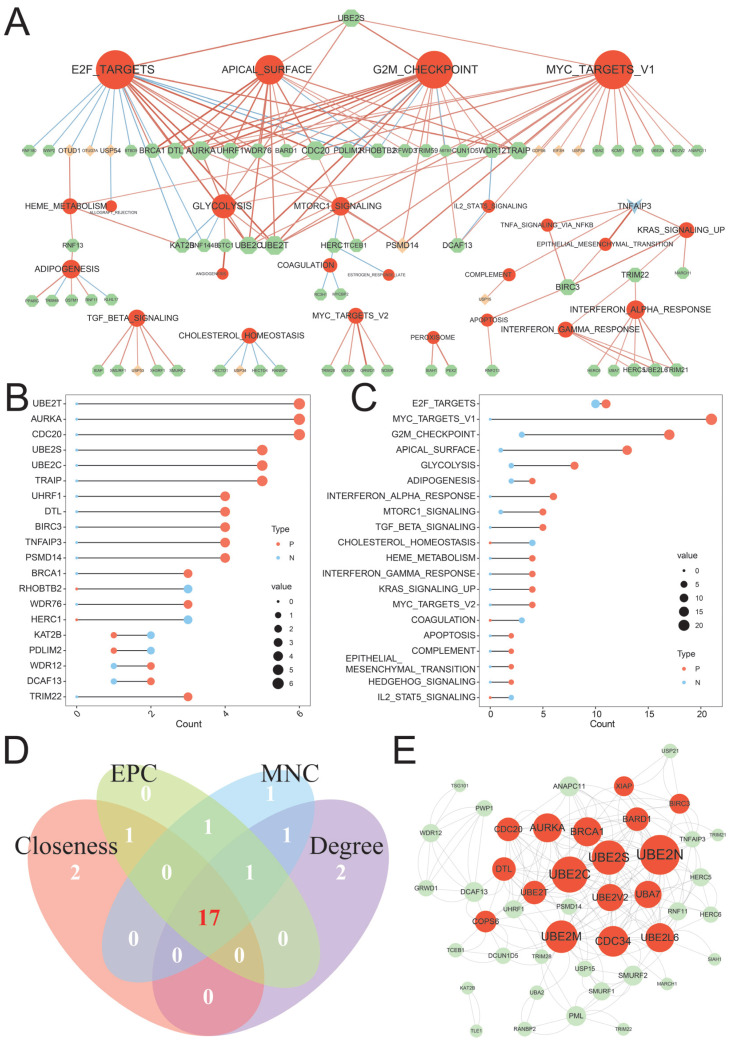
Identification of hub UBRs in LUAD. (**A**) The correlation network diagram of UBRs and cancer hallmark-related pathways. Red nodes represent pathways, green nodes represent writers, light red nodes represent erasers, and light blue nodes represent multis. The size of the points is proportional to the size of the degree. The red edge indicates a positive correlation, and the blue edge indicates a negative correlation. (**B**) The number of cancer hallmark-related pathways associated with specific UBRs. (**C**) Number of UBRs associated with specific cancer hallmark-related pathways. (**D**) Venn diagram of the top 20 hub UBRs identified by the four topology algorithms. (**E**) PPI network diagram of hub UBRs. Red nodes represent hub UBRs.

According to previously published data, a total of 877 UBRs (Supplementary Materials File S2: Table S1) were included in the current study [9,10,11]. To determine the molecular mechanism of UBRs in LUAD, we evaluated the correlation between the expression of UBRs and the activity of cancer hallmark-related pathways. The results showed that the activity of 28 cancer hallmark-related pathways was significantly correlated with the expression of 79 UBRs (Figure 1A; Supplementary Materials File S2: Table S2). Among them, most UBRs belong to the functional category of writers (more than 80%), followed by erasers. In addition, the activity of 23 carcinogenic pathways was closely associated with the expression of multiple UBRs, such as E2F targets, MYC targets V1, G2M checkpoint, and glycolysis (Figure 1B). We also noticed that more than 40% of UBRs were significantly associated with multiple oncogenic pathways, and the expression of most of them was positively correlated with the activity of oncogenic pathways, such as *UBE2T*, *AURKA*, and *CDC20* (Figure 1C).

Then, we used the STRING database to predict the protein–protein interaction (PPI) network of UBRs correlated with hallmark-related pathways (Supplementary Materials File S2: Table S3). Four topological algorithms of EPC, Degree, Maximum Neighborhood Component (MNC) and Closeness are respectively executed to identify the top 20 hub genes from the complex PPI networks. It is interesting to note that 17 UBRs were identified as hub UBRs in all four topological algorithms (Figure 1D,E). Therefore, it is reasonable to believe that these 17 UBRs can be regarded as the hub genes of the ubiquitination network. The above analysis showed that UBRs are correlated with various oncogenic pathways, and 17 hub UBRs in the ubiquitination network were recognized.

### 2.2. Genetic and Transcriptional Alterations of Hub UBRs

**Figure 2:**
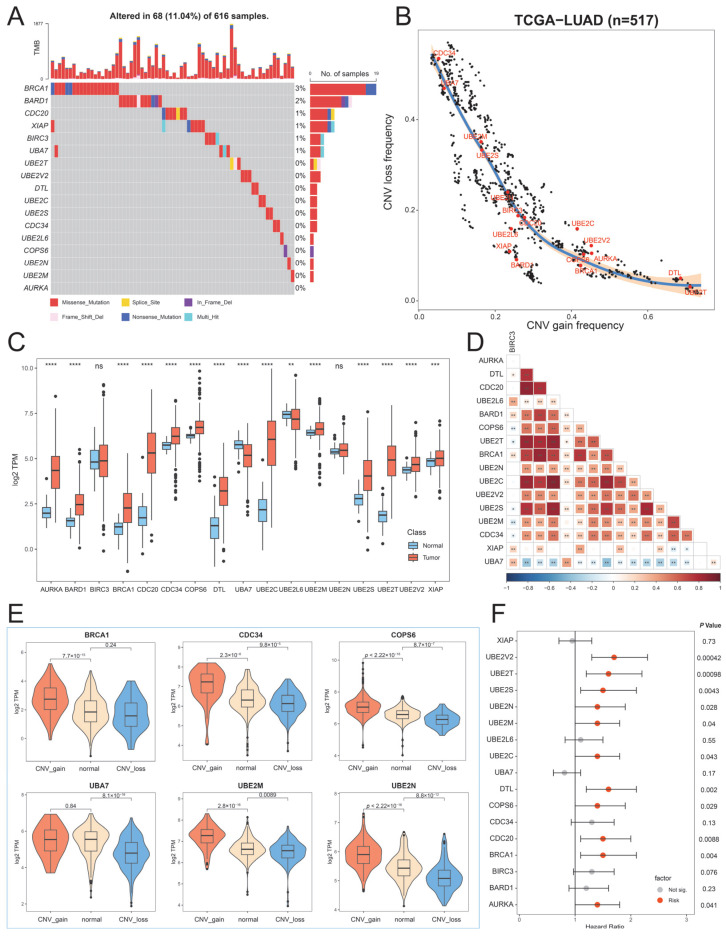
Genetic and transcriptional dysregulation of hub UBRs. (**A**) Waterfall plot of hub UBRs in LUAD. (**B**) CNV frequency distribution of UBRs in LUAD. (**C**) The expression boxplot of hub UBRs in normal and cancer samples. (**D**) Correlation heatmap between hub UBRs. * indicates that the *p*-value is less than 0.05, ** indicates that the *p*-value is less than 0.01, *** indicates that the *p*-value is less than 0.001, **** indicates that the *p*-value is less than 0.0001, and ns indicates a *p*-value greater than 0.05. The color of each square in the heatmap indicates the size of the correlation coefficient. (**E**) The expression of hub UBRs in CNV amplification, CNV loss, and CNV normal samples. (**F**) Forest plot of hub UBRs. The red dot represents the risk factor, and the gray dot indicates no effect.

To explore genetic alterations of UBRs in LUAD, we evaluated the prevalence of non-silent somatic mutations in 17 hub UBRs. Somatic mutations in hub UBRs occurred in 68 of 616 LUAD patients, with a frequency of 11.04%. Among them, *BRCA1* (3%) had the highest mutation frequency, followed by *BARD1* (2%), while *AURKA* did not show any mutation in LUAD patients (Figure 2A). It is worth noting that the mutations of hub UBRs in LUAD are mainly missense mutations. Further investigation of copy number variation (CNV) revealed that there were widespread CNV alterations in UBRs. The hub UBRs, *DTL* and *UBE2T*, exhibit higher frequencies of CNV amplification, while *CDC34* and *UBA7* exhibit higher frequencies of CNV loss (Figure 2B). Subsequently, we investigated the transcriptional changes of hub UBRs in normal and cancer samples. The results showed that except for *BIRC3* and *UBE2N*, other hub UBRs were dysregulated, and most of them were overexpressed in cancer patients (Figure 2C). In addition, the expressions of hub UBRs were highly correlated and mostly positively correlated, suggesting extensive interactions between them (Figure 2D). In order to determine the impact of genetic alterations on the dysregulation of hub UBRs, we investigated the relationship between CNV alterations and gene expression. The results revealed that CNV amplification in hub UBRs led to higher gene expression (e.g., *BRCA1*, *CDC34*, *COPS6*, *UBE2M*, and *UBE2N*), while CNV loss led to lower gene expression (e.g., *UBA7*, *COPS6*, *UBE2M*, and *UBE2N*) (Figure 2E). Then, we further explored the effect of the expression of hub UBRs on the survival of patients. Eleven hub UBRs can affect patients’ survival, and their high expression was detrimental to patient survival (Figure 2F). The above analysis shows the high heterogeneity of hub UBRs in normal and cancer samples and their association in genomics and transcriptomics, suggesting that dysregulation of hub UBRs at the transcriptional level plays an essential role in the occurrence and progression of LUAD.

**Figure 3:**
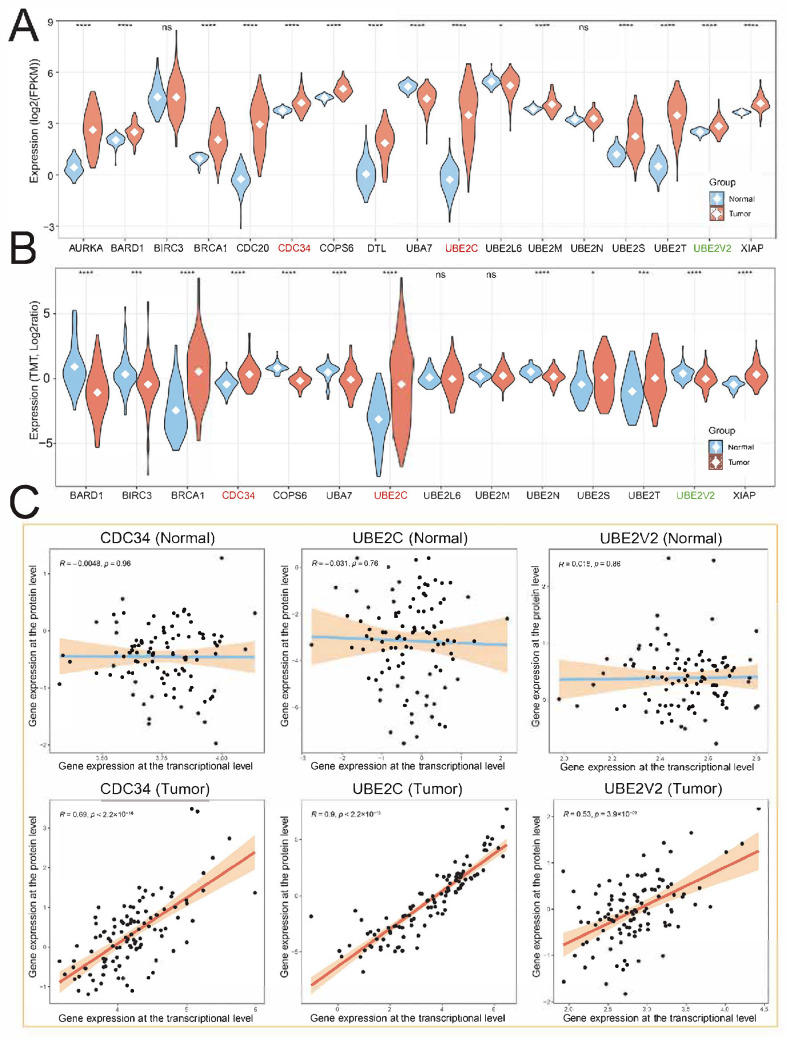
Analysis of transcriptional and protein expression patterns of hub UBRs. (**A**,**B**) Violin plots of hub ubiquitination regulator expression in normal and cancer samples from the CPTAC-LUAD cohort. (**A**) Transcriptional expression; (**B**) protein expression. * represents a *p*-value of less than 0.05, *** represents a *p*-value of less than 0.001, **** represents a *p*-value of less than 0.0001, and ns represents a *p*-value of more than 0.05. (**C**) Scatter plot of the correlation between transcription and protein expression of hub UBRs. Top: normal samples; bottom: cancer samples.

We then evaluated the relationship between transcription and protein levels of hub UBRs in a LUAD cohort from CPTAC (Clinical Proteomic Tumor Analysis Consortium) [21]. The results showed that the transcriptional expression pattern of hub UBRs in the CPTAC-LUAD cohort was similar to that in the TCGA-LUAD cohort. Compared with normal samples, the expression levels of most hub UBRs in cancer were significantly increased (Figure 3A). Analysis of the protein expression pattern of hub UBRs indicated that 14 UBRs could be detected at the protein level, while AURKA, CDC20, and DTL were not expressed at the protein level. Interestingly, the expression patterns of BARD1, COPS6, and UBE2V2 at the protein level are opposite to those at the transcriptional level, and their protein expression levels in cancer are significantly lower than those in normal samples, which may be due to their being affected by post-transcriptional regulation. The protein expression patterns of BRCA1, CDC34, UBA7, UBE2C, UBE2S, UBE2T, and XIAP were consistent with the transcriptional expression patterns, implying that they are good biomarkers in LUAD (Figure 3B). Moreover, the correlation analysis between transcriptional expression and protein expression of hub UBRs showed that after cancer occurrence, except for BIRC3 and COPS6, the absolute values of the Pearson correlation coefficients between transcription and protein expression of other hub UBRs increased (Figure 3C; Supplementary Materials File S1: Figure S1). In summary, the results of this part indicate that the post-transcriptional regulation of hub UBRs is widely weakened after cancer occurrence.

### 2.3. Identification of Ubiquitination Subtypes in LUAD

**Figure 4:**
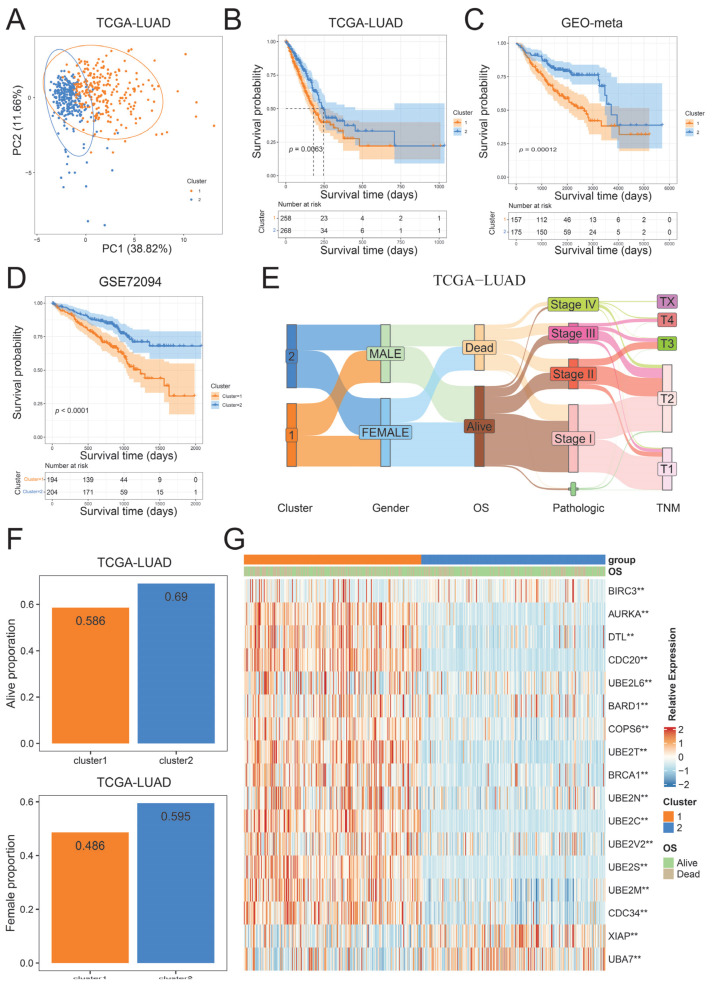
Identification and characterization of ubiquitination subtypes. (**A**) Principal component analysis of ubiquitination subtypes in the TCGA-LUAD cohort. (**B**–**D**) Survival analysis of two ubiquitination subtypes. (**B**) TCGA-LUAD; (**C**) GEO-meta; (**D**) GSE72094. (**E**) Sankey diagram between ubiquitination subtypes and clinical information. (**F**) Survival rate and proportion of female patients in ubiquitination subtypes. (**G**) Expression heatmap of hub UBRs in TCGA-LUAD cohort. ** represents a *p*-value of less than 0.01.

To investigate the impact of hub UBRs on patient classification, we performed consensus cluster analysis based on the expression of 17 hub UBRs. The consensus matrix indicates that the best classification in the TCGA-LUAD cohort is two clusters (Supplementary Materials File S1: Figure S2). Patients were divided into two ubiquitination subtypes based on the classification results, with 266 patients in cluster 1 subtype and 273 patients in cluster 2 subtype. The results of PCA showed that all patients could be broadly classified into two categories, further confirming two different subtypes (Figure 4A). In addition, it was also observed in the GEO-meta and GSE72094 datasets that their best classification cluster was 2, which could also divide patients into two ubiquitination subtypes (Supplementary Materials File S1: Figure S3). Survival analysis of three independent LUAD cohorts found that patients in the cluster 2 subtype had a significantly higher survival advantage than those in the cluster 1 subtype (Figure 4B–D). To explain this phenomenon, we explored the relationship between clinical factors and ubiquitination subtypes in the TCGA-LUAD cohort. Compared with patients in the cluster 1 subtype, patients in the cluster 2 subtype had significantly higher proportions of females and higher survival rates (chi-square test, *p*-value < 0.05) (Figure 4E,F). In addition, 15 hub UBRs were found to be significantly differentially expressed among different ubiquitination subtypes in all three cohorts, among which BIRC3 and UBA7 were highly expressed in cluster 2 subtypes, while *AURKA*, *DTL*, *CDC20*, *BARD1*, *COPS6*, *UBE2T*, *BRCA1*, *UBE2N*, *UBE2C*, *UBE2V2*, *UBE2S*, *UBE2M*, and *CDC34* were highly expressed in cluster 1 subtypes (Figure 4G; Supplementary Materials File S1: Figure S4). The above results indicate that the expression of hub UBRs can be used for the prognostic classification of LUAD, implying that hub UBRs may affect the development of tumors through some potential mechanisms, which in turn leads to differences in the prognosis of patients in various subtypes.

### 2.4. The Immune Landscape of Distinct Ubiquitination Subtypes

**Figure 5:**
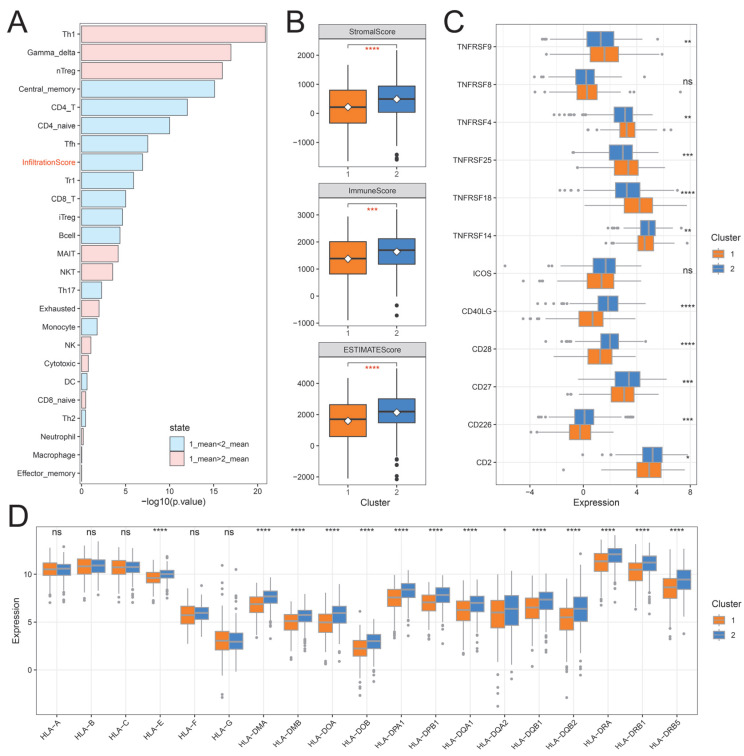
The immune landscape of ubiquitination subtypes in the TCGA cohort. (**A**) Differences in immune cell abundance between ubiquitination subtypes. (**B**) Stromal score, immune score and ESTIMATE score in ubiquitination subtypes. (**C**) Expression of T cell stimulators in ubiquitination subtypes. (**D**) Expression of major histocompatibility complex between ubiquitination subtypes. * means that the *p*-value is less than 0.05, ** means that the *p*-value is less than 0.01, *** means that the *p*-value is less than 0.001, **** means that the *p*-value is less than 0.0001, and ns means that the *p*-value is greater than 0.05.

To explore the potential mechanism of patient survival differences among ubiquitination subtypes, we used the ImmuCellAI (Immune Cell Abundance Identifier, version 0.1.0) tool to study the tumor immune microenvironment between different subtypes. The cluster 2 subtype has a significantly higher infiltration score than the cluster 1 subtype and has different infiltration characteristics between subtypes. The cluster 1 subtype was characterized by high infiltration of Th1, Gamma_delta, nTreg, MAIT, and NKT, while the cluster 2 subtype was characterized by high infiltration of Central_memory, CD4_T, CD4_naive, Tfh, Tr1, CD8_T, iTreg, and Bcell (Figure 5A). We then calculated the tumor-associated stroma (StromaScore), the level of immune cell infiltration (ImmuneScore) and the score of tumor purity (ESTIMATEScore) in the malignant tumor tissue using the ESTIMATE algorithm. Compared with the cluster 1 subtype, the cluster 2 subtype had significantly higher (Wilcoxon rank sum test) stroma score, immune score, and ESTIMATE score (Figure 5B). In addition, we investigated the relationship between subtypes and T cell stimulators and major histocompatibility complex. T-cell stimulators *TNFRSF4*, *TNFRSF9*, *TNFRSF18*, and *TNFRSF25* were highly expressed in the cluster 1 subtype, while *CD2*, *CD266*, *CD27*, *CD28*, *CD40LG*, and *TNFRSF14* were highly expressed in the cluster 2 subtype (Figure 5C). Major histocompatibility complexes, except for *HLA-A*, *HLA-B*, *HLA-C*, *HLA-F,* and *HLA-G*, exhibited no significant difference between subtypes. The rest of the major histocompatibility complex had significantly higher expression levels in the cluster 2 subtype (Figure 5D).

**Figure 6:**
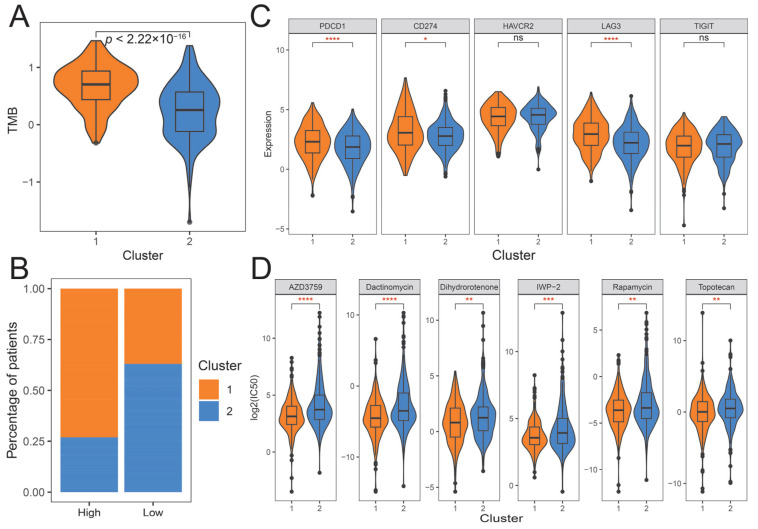
Prediction of ubiquitination subtypes in cancer treatment response. (**A**) Tumor mutation burden score in ubiquitination subtypes. (**B**) The distribution of patients in the tumor mutation burden group. (**C**) Expression of immune checkpoint molecules in ubiquitination subtypes. (**D**) Therapeutic response of ubiquitination subtypes to six drugs. * indicates a *p*-value of less than 0.05, ** indicates a *p*-value of less than 0.01, *** indicates a *p*-value of less than 0.001, **** indicates a *p*-value of less than 0.0001, and ns indicates a *p*-value of greater than 0.05.

Subsequently, we investigated whether there was a significant correlation between ubiquitination subtypes and the efficacy of immunotherapy. The Wilcoxon rank sum test results showed that patients in the cluster 1 subtype had a higher tumor mutation burden than those in cluster 2 subtype (Figure 6A,B). Compared with the cluster 2 subtype, immune checkpoint molecules *PD-1* (*PDCD1*), *PD-L1* (*CD274*), and *LAG3* were expressed at higher levels in the cluster 1 subtype (Figure 6C). In addition, we predicted the difference in drug sensitivity between subtypes by the expression of patients in the TCGA-LUAD cohort. A total of 122 drugs were significantly different between subtypes, of which 108 drugs had higher IC50 values in the cluster 2 subtype, such as AZD3759, Dactinomycin, Savolitinib, Foretinib, and Gallibiscoquinazole, while only 14 drugs had higher IC50 values in cluster 1 subtype, such as Doramapimod and Ribociclib (Figure 6D; Supplementary Materials File S2: Table S4). The analysis of this part reveals the differences between ubiquitination subtypes in the immune map and the effect of therapy, suggesting that the difference in immune response is one of the factors contributing to the difference in patient survival in ubiquitination subtypes.

### 2.5. Construction of the Ubiquitination Score and Its Clinical Characteristics

**Figure 7:**
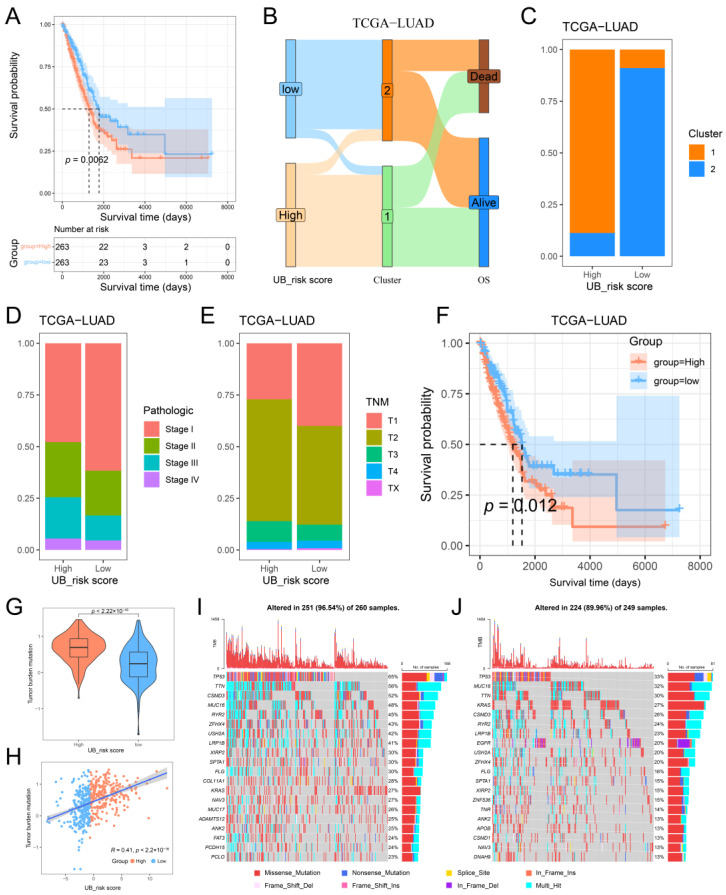
The construction of UB_risk score and its clinical characteristics. (**A**) Survival plot between UB_risk score groups in the TCGA-LUAD cohort. (**B**) Relationship between UB_risk score groups and ubiquitination modification subtypes. (**C**) Distribution of ubiquitination modification subtypes among UB_risk score groups. (**D**) The distribution of pathological stages in the UB_ risk score group. (**E**) The distribution of TNM stage in UB_risk score group. (**F**) Survival curve of the UB_risk score group in the T2-T4 stage of TNM. (**G**) Boxplot of tumor mutation burden between UB_risk score groups. (**H**) Scatter plot of UB_risk score and tumor mutation burden. (**I**,**J**) Waterfall plot of the top 20 frequently somatically mutated genes. (**I**) High-UB_risk score group; (**J**) low UB_risk score group.

Ubiquitination plays a vital role in the occurrence and progression of LUAD. Although the above results have confirmed the value of hub UBRs in LUAD prognostic classification and immune infiltration, these analyses are based on patient populations and cannot predict the ubiquitination subtypes of individual LUAD patients. In view of the heterogeneity and complexity of ubiquitination in patients with LUAD, we constructed a scoring system based on hub UBRs to quantify the ubiquitination pattern of individual LUAD patients, termed the ubiquitination-related risk (UB_risk) score. Subsequently, we divided the patients into high or low ubiquitination groups according to the median of the UB_risk score to evaluate the clinical correlation characteristics of the UB_risk score. Survival analysis showed that the survival rate of patients with a high-UB_risk score was significantly lower than that of patients with a low-UB_risk score (Figure 7A). In addition, we also characterized the relationship between UB_risk scores and ubiquitination subtypes. The high-UB_risk score group was mainly composed of the cluster 1 subtype, while the low-UB_risk score group was mainly composed of the cluster 2 subtype (Figure 7B,C). Analysis of the pathological stage and TNM stage showed that the high-UB_risk score group had more advanced patients compared with the low-UB_risk score group (Figure 7D,E). In addition, compared with the early stages of pathology and TNM, the UB_risk score was significantly increased in the middle and late stages of pathology and TNM, which means that a high-UB_risk score is a risk factor for patients with LUAD (Supplementary Materials File S1: Figure S5). It is worth noting that in the T2-T4 stage of TNM, the prognosis of patients in the high-UB_risk score group was worse than that of patients in the low-UB_risk score group (Figure 7F). Importantly, a similar pattern was found for UB_risk scores constructed in two other independent datasets by the same method. That is, compared with patients in the low-UB_risk score group, patients in the high-UB_risk score group had a worse prognosis (Supplementary Materials File S1: Figure S6), implying the reliability of the UB_risk scoring scheme in the classification of patients with LUAD.

In addition, we also evaluated the distribution of tumor mutation burden in the UB_risk score group. Compared with the group with a low-UB_risk score, the group with a high-UB_risk score had a higher tumor mutation burden, and the UB_risk score was positively correlated with the tumor mutation burden (Figure 7G,H). The list of somatic mutations in the high and low-UB_risk score groups was displayed (Figure 7I,J). The top three genes in the high-UB_risk score group were *TP53* (65%), *TTN* (56%) and *CSMD3* (52%), while the top three genes in the low-UB_risk score group were *TP53* (33%), *MUC16* (32%) and *TTN* (30%). In conclusion, we constructed a scoring scheme to assess the ubiquitination status of individual LUAD patients and revealed its clinical relevance and somatic mutation signature.

### 2.6. Immune Characteristics of UB_Risk Score

**Figure 8:**
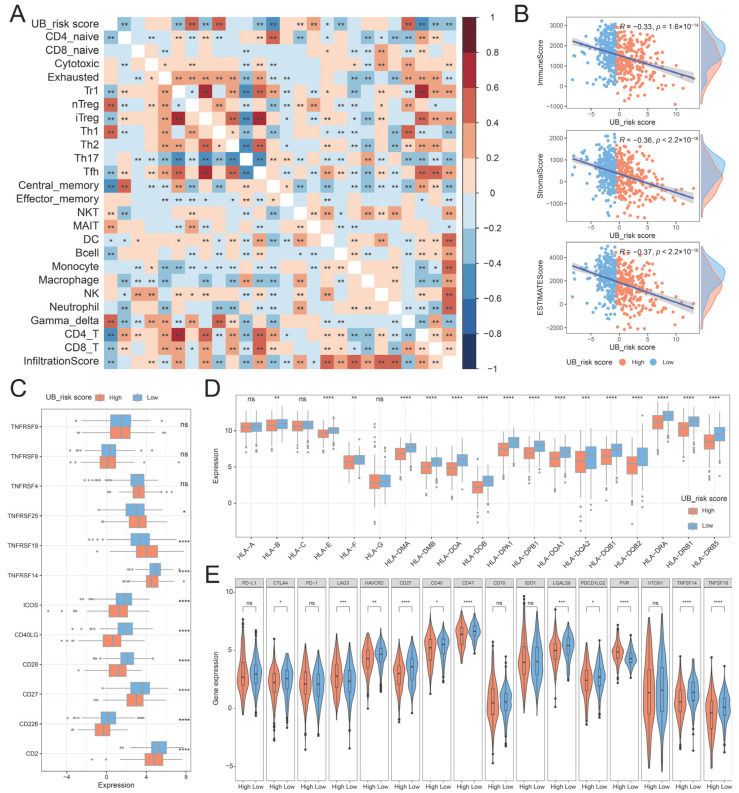
Immune landscape of UB_risk score. (**A**) Correlation heatmap between UB_ risk score and immune cell abundance in the TCGA-LUAD cohort. (**B**) Scatter plot of UB_risk score with stromal score, immune score, and ESTIMATE score. (**C**) The expression of T cell stimulator in the UB_risk score group. (**D**) The expression of major histocompatibility complex in UB_risk score group. (**E**) The expression of immune checkpoint molecules in the UB_risk score group. ns represents that the statistics are not significant. * represents a *p*-value of less than 0.05, ** represents a *p*-value of less than 0.01, *** represents a *p*-value of less than 0.001, **** represents a *p*-value of less than 0.0001, and ns represents a *p*-value of more than 0.05.

To evaluate the relationship between the UB_risk score and the tumor microenvironment, we calculated the correlation between the UB_risk score and tumor immune infiltrating cell abundance. The results showed that the UB_risk score was negatively correlated with the infiltration score and significantly correlated with the abundance of 14 immune cells (Figure 8A). Then, we calculated the correlation between the UB_risk score and StromaScore, ImmuneScore, and ESTIMATEScore. The results showed that the UB_risk score was negatively correlated with StromaScore, ImmuneScore, and ESTIMATEScore, and compared with the high-UB_risk score group, the low-UB_risk score group had a higher StromaScore, ImmuneScore, and ESTIMATEScore (Figure 8B). In addition, we also explored the relationship between UB_risk scores and T cell stimulators and major histocompatibility complexes. The T cell stimulators *TNFRSF18* and *TNFRSF25* had higher expression levels in high-UB_risk score groups, while *CD2*, *CD226*, *CD27*, *CD28*, *CD40LG*, *ICOS*, and *TNFRSF14* had higher expression levels in low-UB_risk score groups (Figure 8C). Interestingly, major histocompatibility complex molecules have higher expression levels in low-UB_risk score groups than in high-UB_risk score groups (Figure 8D).

In addition, the expression levels of immune checkpoints in the high and low-UB_risk score groups were examined. *LAG3* and *PVR* exhibited higher expression in the high-UB_risk score group, while *CTLA4*, *HAVCR2*, *CD27*, *CD40*, *CD47*, *LGALS9*, *PDCD1LG2*, *TNFSF14* and *TNFSF18* showed higher expression in the low-UB_risk score group (Figure 8E). Immunophenscore (IPS) analysis showed that the IPS of the low-UB_risk score group was significantly higher than that of the high-UB_risk score group, hinting that patients in the low-UB_risk score group were more likely to obtain clinical benefits from immunotherapy (Supplementary Materials File S1: Figure S7). In summary, the results of this part illustrate the immune characteristics of the UB_risk score, and patients with low-UB_risk scores are more likely to benefit from immunotherapy.

## 3. Discussion

Protein ubiquitination is involved in a diverse range of biological processes, such as proteasome protein degradation, signal transduction, DNA repair, gene transcription, and kinase activation. It is a dynamic and reversible post-translational modification mediated by UBRs [22,23,24]. Ubiquitination represents a general regulatory mechanism of signal transduction. Dysregulation of ubiquitin signaling is associated with the occurrence and progression of a variety of human diseases, including cancer. Targeting UBRs has evolved as an effective strategy for therapeutic intervention [13,25]. For example, Ubiquitin-conjugating enzyme E2 is dysregulated in many cancers and is involved in a variety of tumor-promoting processes, including DNA repair, apoptosis, cell cycle progression, and oncogenic signaling pathways. It is a potential biomarker for cancer diagnosis, treatment, and prognosis [26]. The UBE2C/CDH1/DEPTOR axis forms an oncogene and tumor suppressor cascade regulating autophagy and cell cycle, and UBE2C is a lung cancer target associated with Kras mutations [27]. Genetic and epigenetic alterations of ubiquitin ligases E3s play an integral role in the occurrence, progression, and treatment of human cancers and are prospective targets for cancer therapy [28,29,30]. Furthermore, in the past few decades, a large number of drugs or inhibitors targeting UBRs (such as *TRAF6*, *LUBAC*, *USP1*, *USP2*, *USP7*, *USP8*, and *USP9X*) have been developed, and some drugs or inhibitors targeting UBRs, such as thalidomide, pomalidomide, and lenalidomide, have been approved by the FDA for the treatment of cancer [30]. Therefore, a comprehensive exploration of the oncogenic pathways, genetic alterations, patient classification, survival prognosis, and therapeutic value of UBRs in LUAD can provide guidance at the ubiquitination level for personalized treatment of LUAD patients.

This study revealed significant correlations between 28 cancer hallmark-related pathways and 79 UBRs, and 17 hub UBRs were identified from the filtered PPI network using four different topological algorithms. These hub UBRs were closely connected. Notably, hub UBRs were widely dysregulated in LUAD, of which 11 hub UBRs had expressions that could affect patient survival, and their high expressions were detrimental to the survival of LUAD patients, while their expressions were mostly affected by CNV amplification or loss. The results of this study are consistent with many previous studies. For instance, *CDC20* was discovered half a century ago and its role in regulating cell cycle progression was elucidated. Cells with *CDC20* mutants prevent cell division, as well as prevent cell cycle progression to the anaphase and chromosome separation. A series of follow-up studies have demonstrated that it plays different biological functions in apoptosis, ciliary disintegration, and brain development. A large amount of evidence shows that *CDC20* plays a carcinogenic role in human tumors and is overexpressed in many types of cancers, such as lung cancer, breast cancer, pancreatic cancer, prostate cancer, and colorectal cancer. It is also a biomarker or potential target for many cancers such as lung cancer, colorectal cancer, and pancreatic cancer [31,32]. *UBE2T* expression is upregulated in LUAD, and this upregulation is associated with tumor growth, lymph node metastasis, distant metastasis, and poor prognosis. It can promote lung adenocarcinoma proliferation by regulating IL-6/STAT3, p53/AMPK/mTOR and Wnt/β-catenin signaling pathways [33]. Knockdown of *CDC34* can inhibit the growth and survival of lung cancer cells, while overexpression can promote the growth and survival of lung cancer cells. Studies have shown that *CDC34* can positively regulate EGFR-mediated oncogenic signals, thereby stabilizing EGFR [34].

The hub UBRs-based expression matrix found two ubiquitination subtypes with different characteristics in LUAD patients. In three independent datasets, it was found that patients in the cluster 2 subtype had a survival advantage over patients in the cluster 1 subtype. This advantage may be due to multiple factors such as the lower tumor mutation burden, the higher proportion of females, and the weaker expression perturbations in patients in the cluster 2 subtype. The tumor microenvironment is composed of many different cells (such as cancer cells, stromal cells, blood vessels, nerve fibers, extracellular matrix) and non-cellular components. It drives tumor growth, invasion, metastasis, and the therapeutic response. It is a bridge connecting cancer and the entire organism and plays an essential role in clinical outcomes and therapeutic response [22,35,36]. Further immune signature analysis showed that the ubiquitination subtypes had different infiltration characteristics. The Cluster 1 subtype was characterized by high infiltration of Th1, γδT, nTreg, MAIT and NKT, while the cluster 2 subtype was characterized by high infiltration of Tcm, CD4_T, CD4_naive, Tfh, Tr1, CD8_T, iTreg, and B cells. Most T cell stimulators, major histocompatibility complex, immune checkpoint molecules, and drug sensitivities were significantly different across ubiquitination subtypes, suggesting the potential clinical value of ubiquitination subtypes in the formulation of LUAD treatment plans.

To assess the ubiquitination status and isoforms of individual LUAD patients, we constructed a UB_risk scoring scheme with stability across datasets based on the expression profiles of hub UBRs. The results of survival analysis demonstrated that patients in the high-UB_risk score group had a worse prognosis than those in the low-UB_risk score group. Further analysis found that tumor mutation burden, immune cell abundance, stromal score, immune score, ESTIMATE score, T cell stimulator, major histocompatibility complex, and immune checkpoints differed significantly between UB_risk score groups. Remarkably, patients with low-UB_risk scores are more likely to benefit from immunotherapy than those with high-UB_risk scores. In addition, we compared the similarities and differences between this study and previous similar studies [37,38,39,40]. First, the genesets used in this study to construct the score are different from those used in previous studies. The geneset used in this study to construct the score is the hub UBRs that regulates the ubiquitination process, while the geneset used by other studies to construct the score comprises ubiquitination-related genes or partial UBRs. Second, the methods of hub gene selection are different. This study uses a network-based approach, while other studies mostly use lasso cox regression models. Third, there are also differences in the score construction methods of different studies. It is worth noting that although there is diversity in the genesets, hub gene selection, and score construction methods used in different studies, all studies have shown that patients in the high-score group often have a worse prognosis than those in the low-score group. The results of this study can provide guidance for the development of precise treatment and immunotherapy strategies for LUAD patients at the ubiquitination level in the future.

This study revealed the clinical relevance and immunological characteristics of the ubiquitination status of patients with LUAD based on UBRs. It provided new insights for understanding the association between the ubiquitination status of patients with LUAD and the tumor microenvironment and immunotherapy. However, this study still needs to be further improved in some respects. For example, single-cell sequencing technology can be integrated to explore the differences in the tumor immune microenvironment, cell communication of LUAD patients with different ubiquitination states at the cellular layer. Furthermore, although this study revealed differences in the treatment responses of LUAD patients with different ubiquitination status or subtypes, the predictive results of drug sensitivity analysis should still be used with caution and need to be validated by experiments.

## 4. Materials and Methods

### 4.1. The Source of UBRs

Based on recently published literature, we collected 877 UBRs in humans, including 603 writers, 103 erasers, 147 readers, and 24 multi [9,10,11] (Supplementary Materials File S2: Table S1). Multi means that the UBR can play multiple functions, e.g., *BIRC6* can serve as a ubiquitin-conjugating enzyme (E2) and an E3 ubiquitin-ligating enzyme (E3).

### 4.2. Collection and Processing of Publicly Available LUAD Cohort Datasets

The expression matrix of LUAD patients with survival information was derived from The Cancer Genome Atlas (TCGA) and Gene Expression Omnibus (GEO) databases. Among them, the expression matrix of the TCGA-LUAD cohort was obtained from the Genomic Data Commons (GDC) through the R package TCGAbiolinks (version 2.26.0) [41]. TCGAbiolinks is a bioinformatics tool that can access The National Cancer Institute (NCI) GDC and allow users to query, search, download and perform integrative analyses. Raw CEL files for GSE31210, GSE37745 and GSE72094 with clinical information were downloaded from the GEO database. Background correction and quantile normalization were performed using the robust multichip average (RMA) algorithm in the R package affy (version 1.76.0). In particular, GSE31210 and GSE37745 were derived from the same sequencing platform (Affymetrix Human Genome U133 Plus 2.0 Array), and batch effects between the two datasets were removed by using the combat method in the sva package (version 3.46.0) [42]. The dataset after removing the batch effect is called GEO-meta. The clinical information of the TCGA-LUAD cohort was downloaded from the GDC TCGA LUAD cohort of UCSC XENA, while the clinical information of GSE31210, GSE37745 and GSE72094 was downloaded from the GEO database.

### 4.3. Gene Set Enrichment Analysis (GSVA) and Identification of Hub Genes

To investigate cancer hallmark-related pathways in which UBRs are involved, we performed gene set enrichment analysis (GSVA) using the GSVA (version 1.46.0) package based on the expression matrix of UBRs in the TCGA-LUAD cohort. GSVA converts the data from the gene of the sample matrix to the gene set of the sample matrix through a non-parametric, unsupervised method to evaluate the pathway enrichment of each sample [43]. The cancer hallmark-related pathways gene set used in GSVA was derived from the Molecular Signatures Database (MSigDB) [44]. Subsequently, we calculated the Pearson correlation coefficient (PCC) between UBR expression and cancer hallmark-related pathway activity to establish the relationship between UBRs and pathways. UBR–pathway pairs with PCC greater than |0.5| and corrected *p* value less than 0.01 were considered to be significantly correlated for further analysis. Cytoscape (version 3.10.3) was used to visualize the UBR–pathway relationship. Based on the STRING interaction database, a protein–protein interaction (PPI) network of UBRs with significantly related pathways was constructed, and edges with interaction scores of less than 0.7 were removed. Then, the PPI network was imported into Cytoscape, and the top 20 hub genes were extracted through four topological algorithms in Cytoscape’s cytoHubba plug-in, including Degree, Closeness, Edge Percolated Component (EPC) and Maximum Neighborhood Component (MNC) [45]. The UBR shared by the four algorithms was regarded as the hub UBR of the entire PPI network. In addition, the Wilcoxon rank sum test was used to analyze whether there were significant differences in hub UBRs between normal and cancer samples in the TCGA-LUAD cohort, and the Spearman correlation coefficient between hub UBRs was calculated through the Hmisc (version 4.8.0) package.

### 4.4. Analysis of Genetic Alterations

The somatic mutations of the TCGA-LUAD cohort were acquired from the GDC database via the R package TCGAbiolinks, and the mutation information was visualized using Maftools (version 2.14.0) [41,46]. The tumor mutation burden information of LUAD patients was obtained using the tmb function of the R package Maftools. Copy number alteration data for the TCGA-LUAD cohort were downloaded from the UCSC Xena platform. The relationship between copy number alterations and expression of hub UBRs was determined by the Wilcoxon rank sum test.

### 4.5. Consensus Clustering Analysis of Hub UBRs

Consistent clustering analysis was performed based on the expression of hub UBRs in different cohorts of cancer patients, utilizing the unsupervised clustering PAM method to perform this procedure through the R package ConsensusClusterPlus (version 1.62.0), and duplicated 2000 times to ensure category stability [47]. To evaluate the distribution differences and clinical value between different ubiquitination subtypes, we executed principal component analysis (PCA) and compared the survival analysis differences between subtypes. The relationship between ubiquitination subtypes and other clinicopathological features (e.g., gender, survival status, pathological stage, etc.) was visualized using the R package ggSankey (version 0.0.99999). The expression pattern heatmap of hub UBRs in ubiquitination subtypes was drawn by ComplexHeatmap (version 2.14.0), and the Wilcoxon rank sum test was used to analyze the expression differences of hub UBRs between ubiquitination subtypes [48].

### 4.6. Analysis of Immune Characteristics of Ubiquitination Subtypes

To assess the differences in immune infiltration among different ubiquitination subtypes, we estimated the abundance of 24 immune cell types by Immune Cell Abundance Identifier (ImmuCellAI) based on the expression profile data of LUAD patients. ImmuCellAI is a gene set signature method that can be used to predict the abundance of 18 T cell types and 6 important immune cells from gene expression data. Among them, 18 T cell types included CD4^+^, CD8^+^, CD4^+^ naïve, CD8^+^ naïve, central memory T (Tcm), effector memory T (Tem), Tr1, iTreg, nTreg, Th1, Th2, Th17, Tfh, Tc, MAIT, Tex, gamma delta T (γδT), and natural killer T (NKT) cells; and six immune cells included B cells, macrophages, monocytes, neutrophils, DC, and NK cells [49]. Subsequently, the stromal score, immune score and estimate score were predicted using the ESTIMATE (version 1.0.13) tool based on the expression profile data of the LUAD cohort. The stromal score reflects the presence of stroma in the tumor, the immune score represents the infiltration of immune cells, and the estimate score reflects the purity of the tumor [50]. To demonstrate the immune characteristics of ubiquitination subtypes, we compared the expression of T cell stimulators, major histocompatibility complex, and immune checkpoint genes between different ubiquitination subtypes. Moreover, the tumor mutation burden between different ubiquitination subtypes (TCGA-LUAD cohort) was also compared. According to the expression profile of LUAD patients, R package oncoPredict (version 1.2) was used to predict the semi-inhibitory concentration (IC50) of chemotherapeutic drugs in each patient, and the difference in drug concentration among ubiquitination subtypes was compared [51,52].

### 4.7. Construction of Ubiquitination-Related Risk Scores

We established a ubiquitination-related risk (UB_risk) score by performing principal component analysis to measure the ubiquitination pattern of individual patients. Specifically, the expression of hub UBRs in the LUAD cohort was normalized, and then principal component analysis (PCA) was executed according to the normalized expression profile of hub UBRs, and principal components 1 and 2 were extracted as signature scores. The highlight of this method is that the scores can be concentrated on the set with the largest block of well-correlated or inverse-correlated genes in the set, and the weight contribution from genes that do not track with other set members can be reduced. A similar formula was used to define the ubiquitination-related risk score: Ub_risk score = ∑(PC1_i_ + PC2_i_), where i represents the expression of hub UBRs [53,54,55].

### 4.8. Survival and Statistical Analysis

The survival risk (hazard ratio, HR) of each hub UBR in LUAD was evaluated by the Cox proportional hazards regression model. The Kaplan–Meier log-rank test of survival was used to statistically estimate whether the survival differences between subtypes/groups were significant, and the R package survminer (version 0.5.0) was used to construct the survival curves between subtypes/groups. All analyses in this study were performed in R software (version 4.2.3). The information about the R packages used is mentioned above. The statistical method for the significance between subtypes or groups was the Wilcoxon rank sum test, and a *p*-value less than 0.05 was considered statistically significant.

## Data Availability

The datasets used in this study are from public databases, and the dataset numbers and processing methods are described in detail in Section 3.

## Supplementary Materials

The following supporting information can be downloaded at https://www.mdpi.com/article/10.3390/ijms26136501/s1.
