# Supplementary material for: Integrative Multi-Omics Analysis Reveals the Molecular Characteristics, Tumor Microenvironment, and Clinical Significance of Ubiquitination Mechanisms in Lung Adenocarcinoma
Source: Int J Mol Sci. 2025 Jul 6;26(13):6501. doi: 10.3390/ijms26136501 (PMC12250011; doi:10.3390/ijms26136501)
Supplement: Supplementary file 1 [file ijms-26-06501-s001.zip › Supplementary File S1-Figures S1-S7.pdf]

Integrative Multi-Omics Analysis Reveals the Molecular Characteristics, Tumor Microenvironment, and Clinical Significance of Ubiquitination Mechanisms in Lung Adenocarcinoma

Deyu Long [1,2,†], Yajing Xue [1,†], Xiushi Yu [1,2,], Xue Qin [1], Jiaxin Chen [1], Jia Luo [1], Ketao Ma [1,2,], Lili Wei [1,2,*] and Xinzhi Li [1,*]

1 The Key Laboratory of Xinjiang Endemic and Ethnic Diseases, Ministry of Education, Shihezi University Medical College, Shihezi 832000, China; maketao@shzu.edu.cn (K.M.)
2 Department of Physiology, Shihezi University School of Medicine, Shihezi 832003, China
* Correspondence: wll1126@shzu.edu.cn (L.W.); lixinzhi@shzu.edu.cn (X.L.)
† These authors contributed equally to this work.

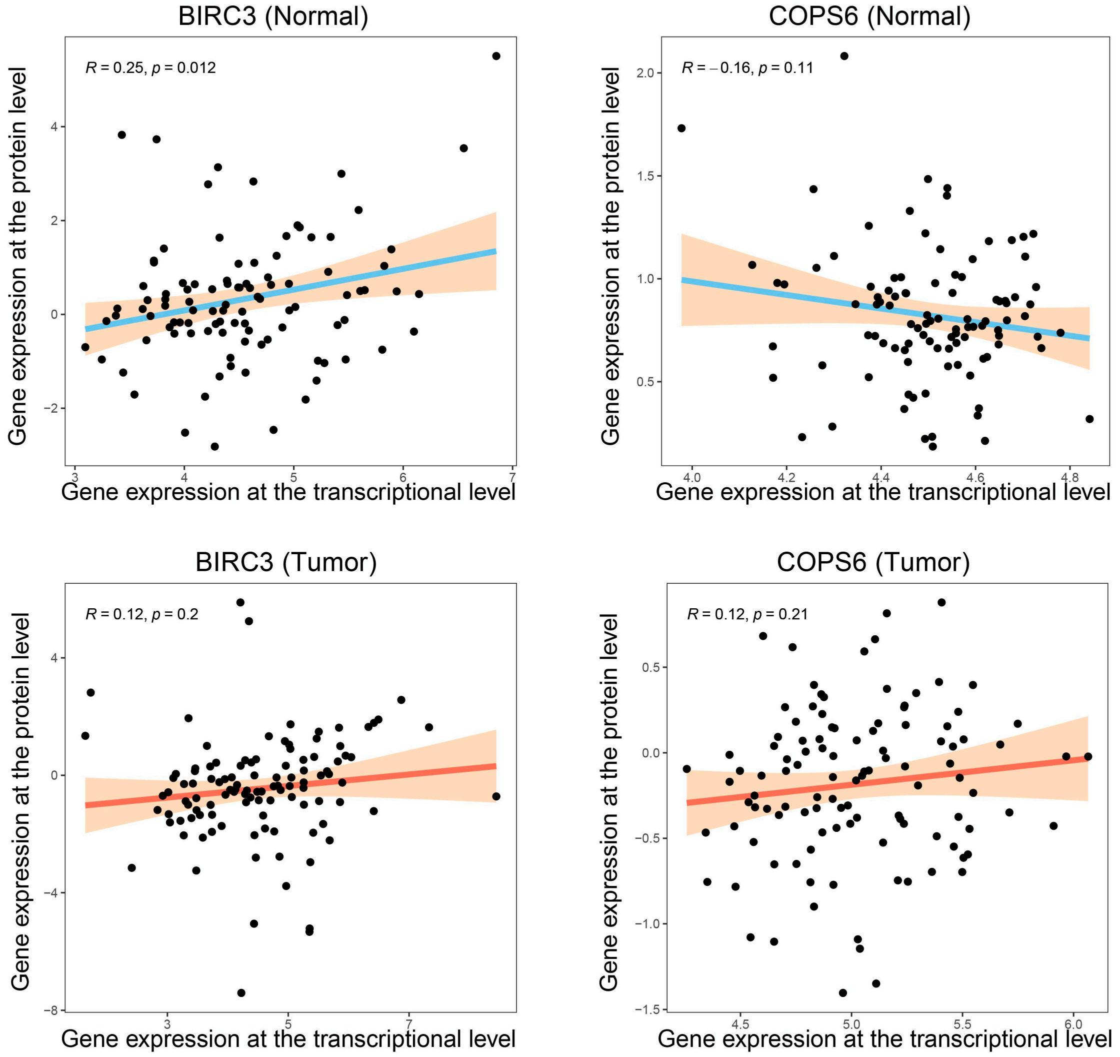

Figure S1. Scatter plot of the correlation between transcription and protein expression of hub UBRs. Top: normal samples; Bottom: cancer samples.

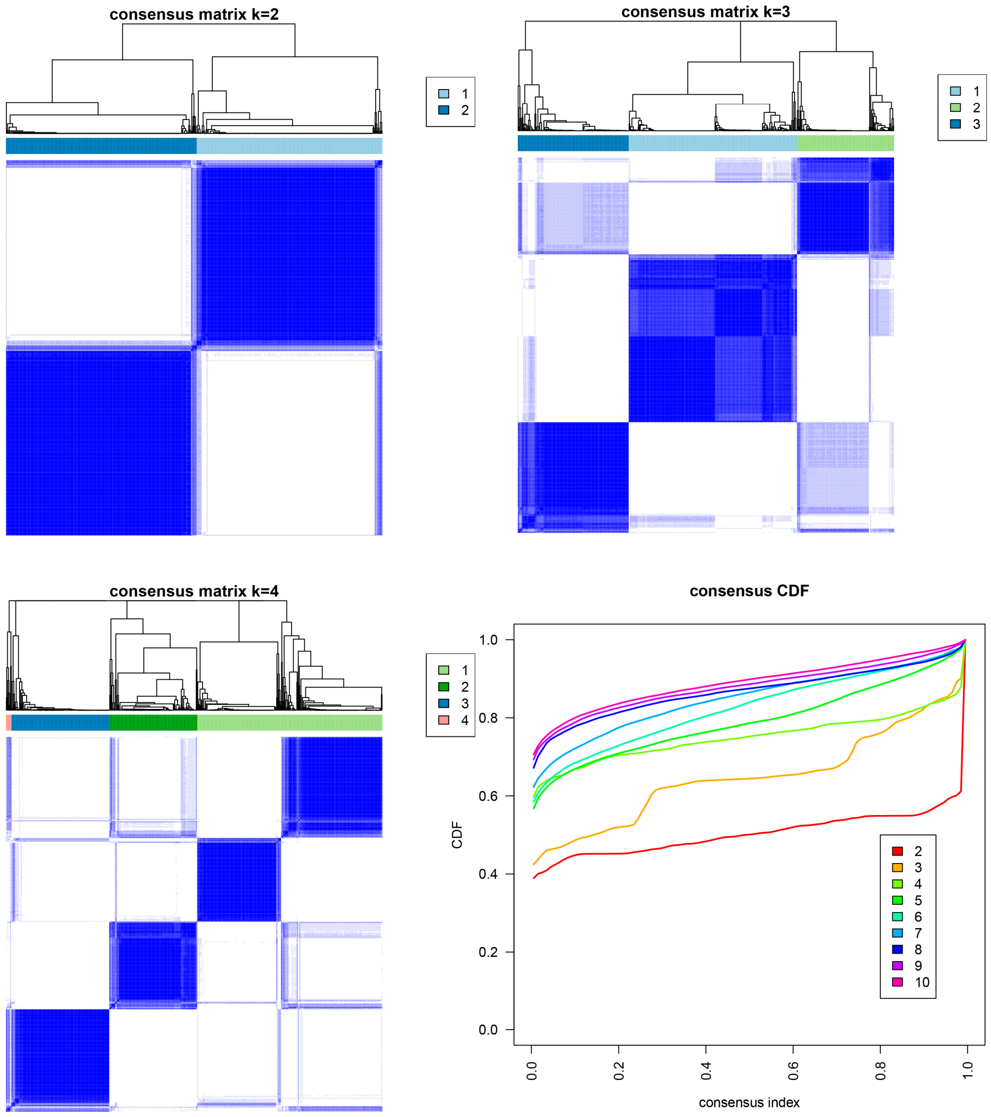

Figure S2. Consistency matrix and cumulative distribution function diagram of TCGA-LUAD cohort.

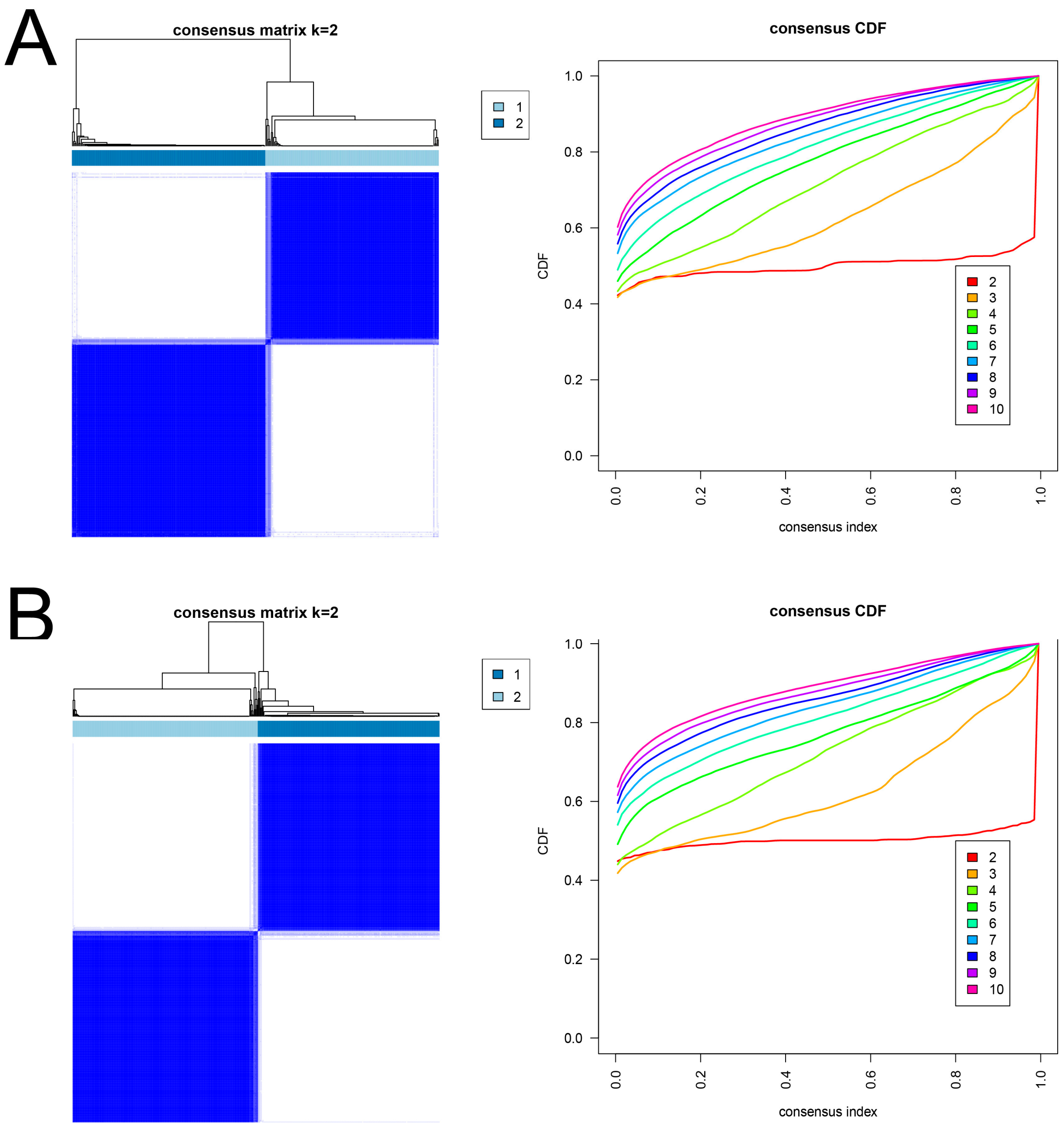

Figure S3. The consistency matrix and cumulative distribution function diagram of the dataset. (**A**) GEO-meta cohort; (**B**) GSE72094 cohort.

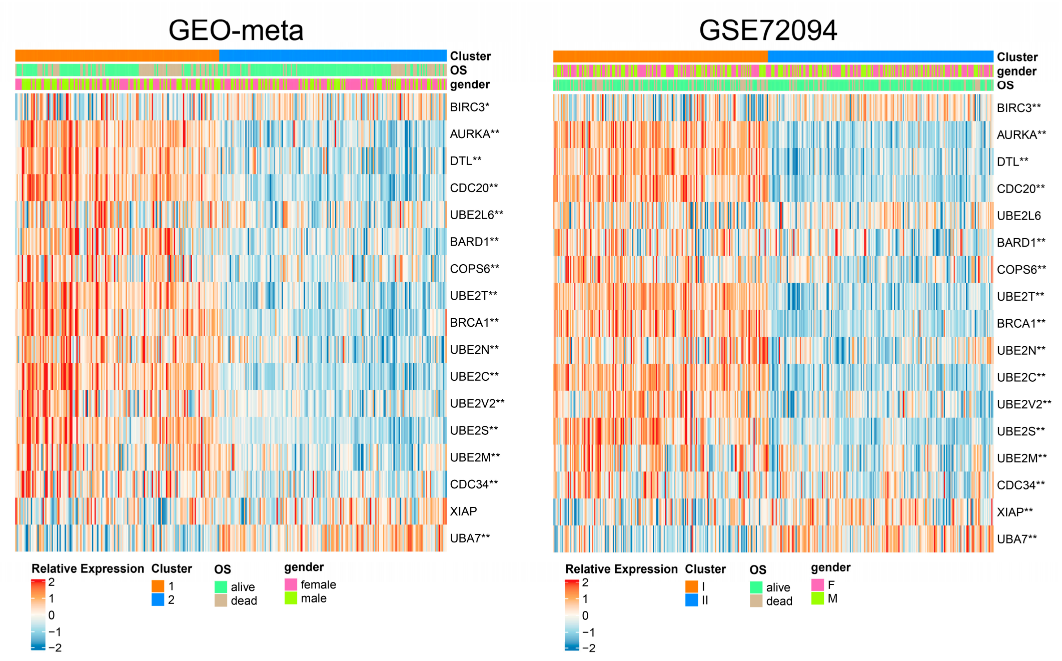

Figure S4. Expression heatmap of hub UBMEs. * represents a *p*-value of less than 0.05, and ** represents a *p*-value of less than 0.01.

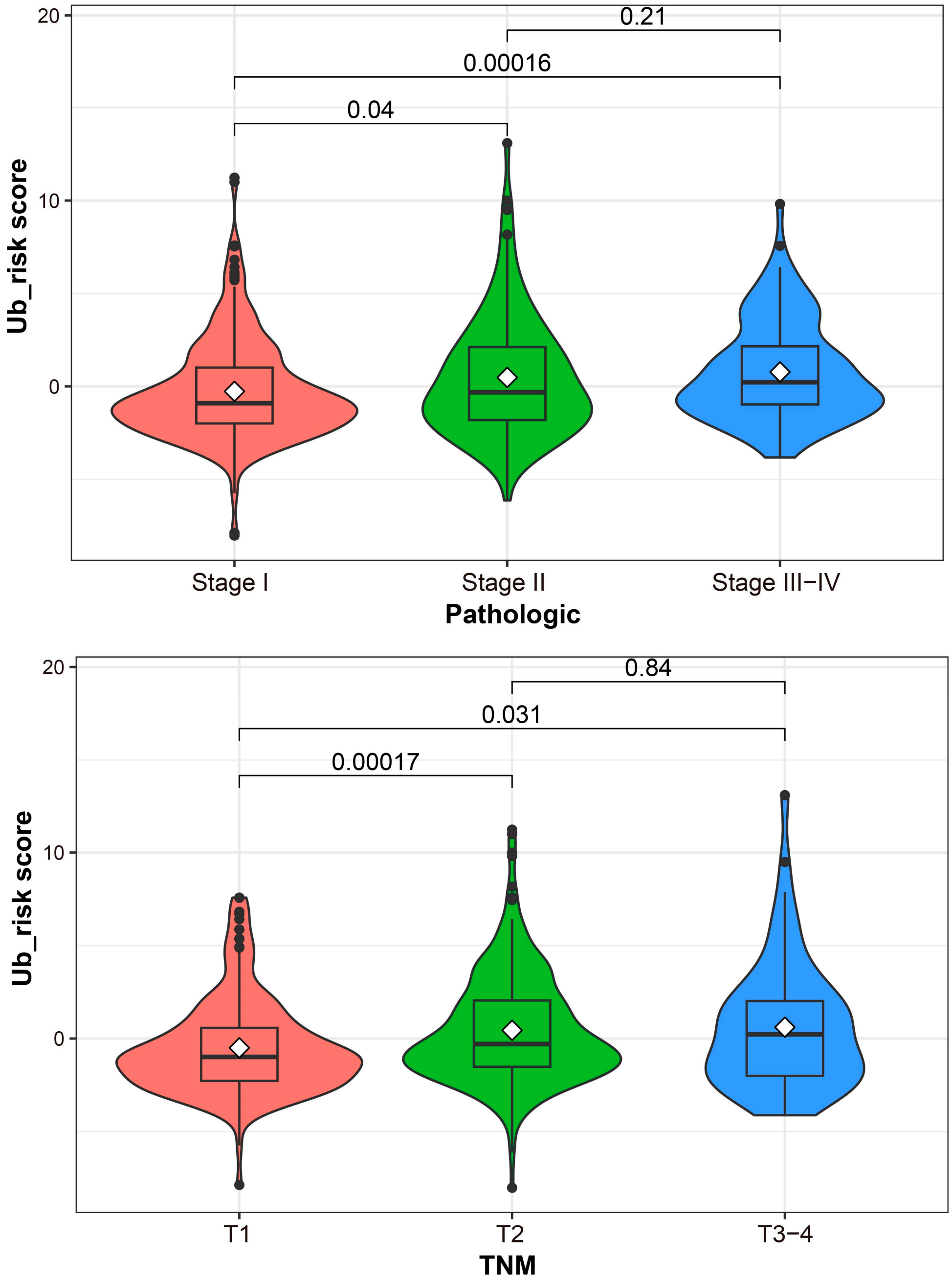

Figure S5. Distribution of UB_risk scores in pathological stage and TNM stage. Top: pathologic stage; bottom: TNM stage.

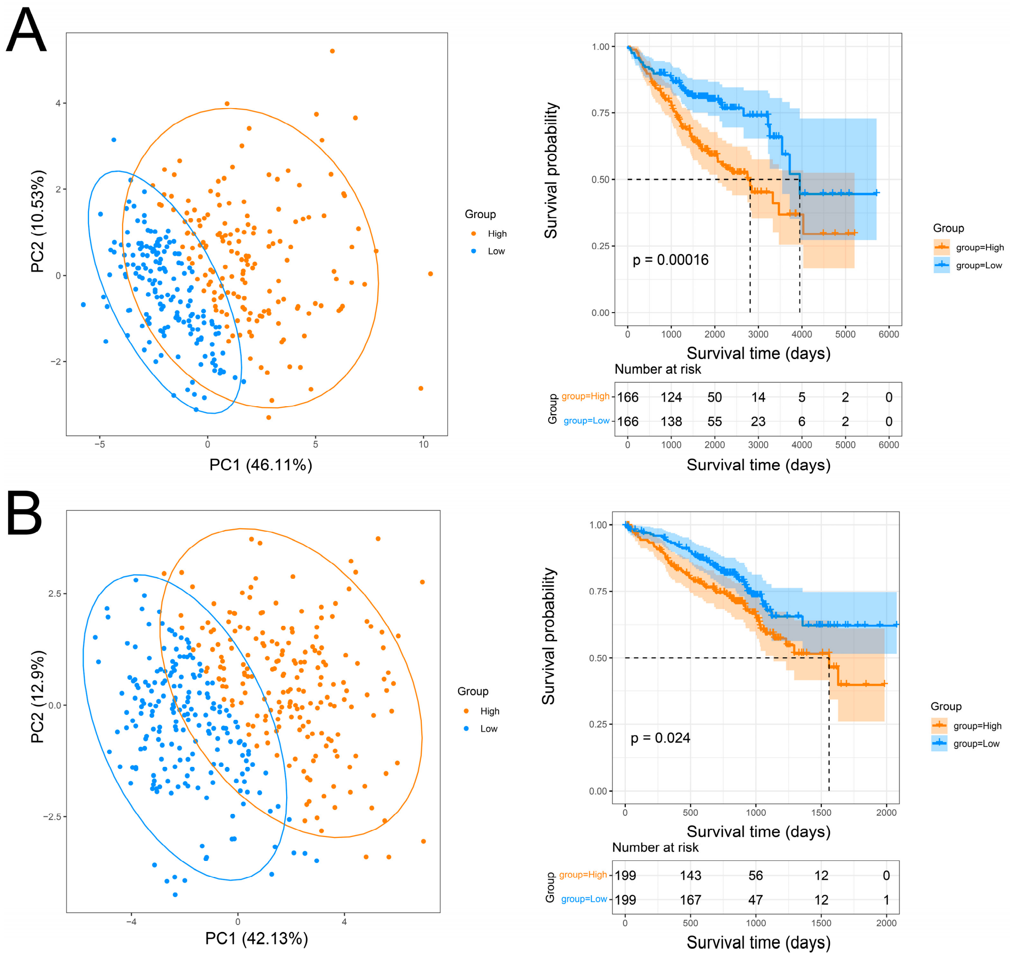

Figure S6. Principal component analysis and survival plots of UB_risk score groups in the LUAD cohort. (**A**) GEO_meta cohort; (**B**) GSE72094 cohort.

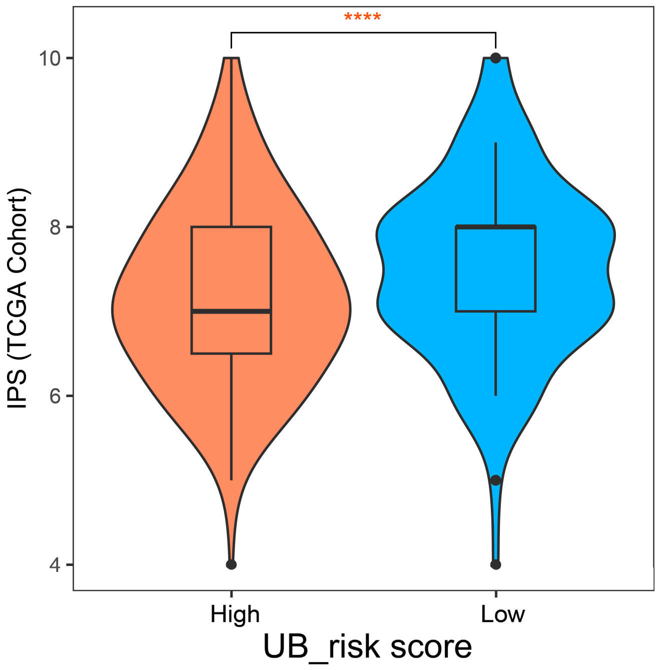

Figure S7. The distribution of IPS scores between UB_risk score groups in the TCGA-LUAD cohort. **** represents a p-value of less than 0.0001.
